# Review on grain size effects on thermal conductivity in ZnO thermoelectric materials

**DOI:** 10.1039/d1ra06133j

**Published:** 2022-02-15

**Authors:** S. Sulaiman, S. Izman, M. B. Uday, M. F. Omar

**Affiliations:** School of Mechanical Engineering, Faculty of Engineering, Universiti Teknologi Malaysia Skudai 81310 Johor Malaysia; Faculty of Manufacturing and Mechatronic Engineering Technology, Universiti Malaysia Pahang Pekan 26600 Pahang Malaysia surayas@ump.edu.my; Centre for Advanced Composite Materials (CACM), Institute for Vehicle Systems and Engineering, Universiti Teknologi Malaysia Skudai 81310 Johor Malaysia; Physics Department, Faculty of Science, Universiti Teknologi Malaysia Skudai 81310 Johor Malaysia

## Abstract

Thermoelectric materials have recently attracted a lot of attention due to their ability to convert waste heat into electricity. Based on the extensive research in this area, the nanostructuring approach has been viewed as an effective strategy for increasing thermoelectric performance. This approach focuses on the formation and growth of the superfine, pure and uniform grain size. Since the grain size has a strong influence on the thermal conductivity, this can be reduced by increasing the phonon scattering at grain boundaries and refining the grain sizes. Therefore, this review aims to discuss the mechanism of reduction in thermal conductivity in small-grain zinc oxide (ZnO) and the optimization techniques for obtaining ZnO nanoparticles with desirably low thermal conductivity and excellent thermoelectric performance.

## Abstract 2

Use of nanomaterials for various energy applications, such as energy generation, conversion, storage, saving and transmission.

## Graphical abstract

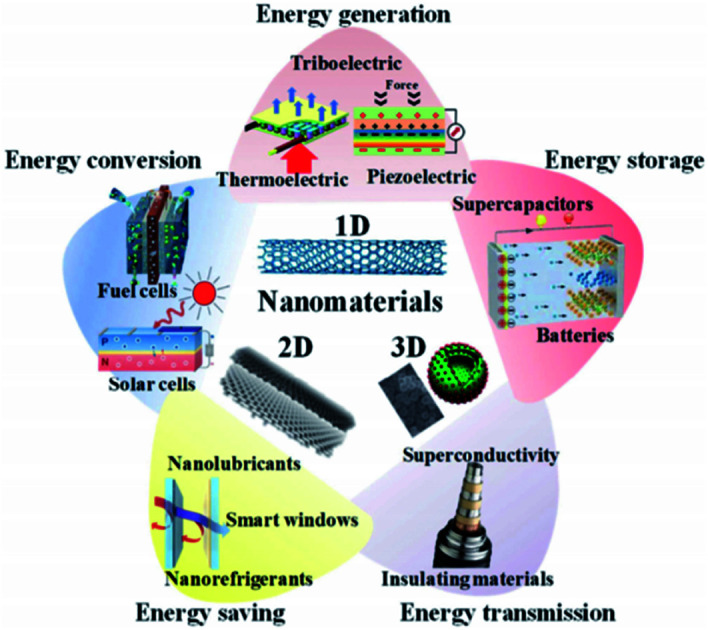

## 1. Introduction

**Fig. 1:**
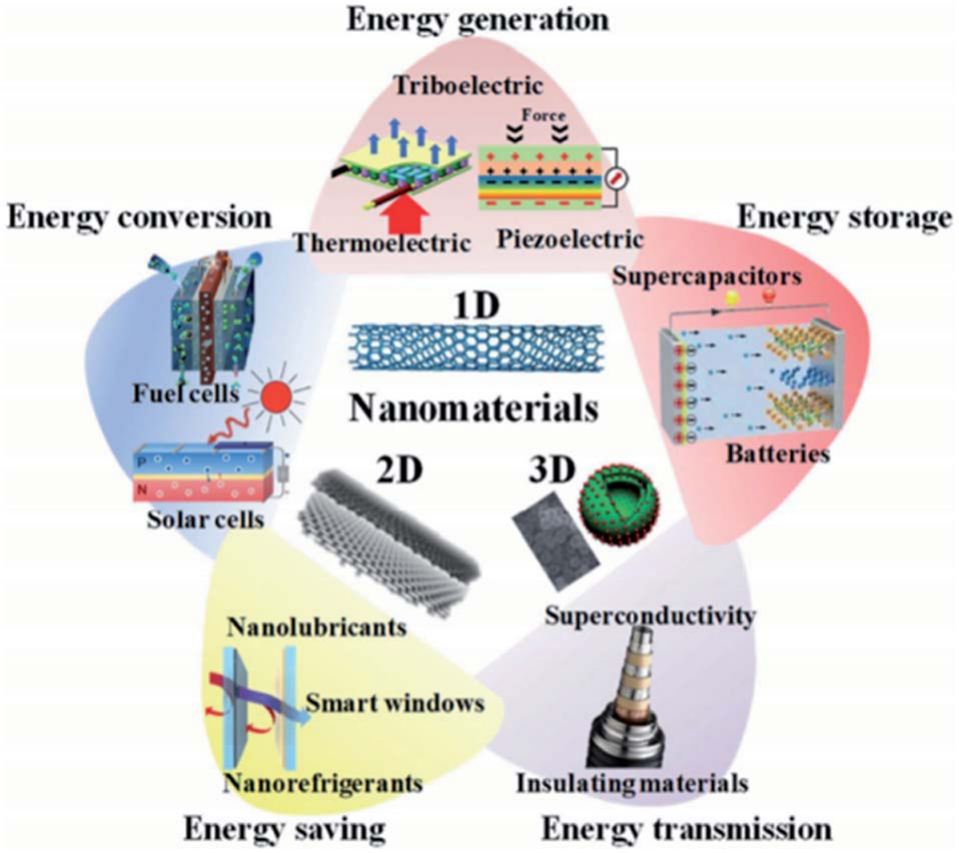
Use of nanomaterials for various energy applications, such as energy generation, conversion, storage, saving and transmission.^4^

The global energy crisis and environmental issues have been prominent in recent years.^1^ Due to the increasing demand for energy and massive oil consumption, an extensive increase in carbon footprint from various industrial processes has been reported.^2^ The processes include the use of coal, natural gas and other non-renewable energy sources in fossil fuel burning, power generation and industrialization. As a result, the greenhouse effects have worsened, causing a catastrophic impact on the environment and our climate. It is predicted that oil and gas reserves will decline over the next 40 years. By 2025, carbon dioxide emissions into the atmosphere are expected to double compared to 1990 levels.^3^ To minimize these problems, various efforts have been made to find alternative sources of energy (Fig. 1). Energy conversion technologies are one of the alternatives. Technologies such as fuel cells and solar cells produce clean energy with zero emissions and are more efficient compared to conventional combustion technologies.^4^ To reduce the dependency on fossil fuels, the thermal source is preferred as alternative energy. As thermal energy can be sourced from solar, automobiles and waste heat from the industries, it incurs minimal cost. It is estimated that around two-thirds of this source is wasted worldwide in the form of waste heat without economic profit.^5^ Therefore, using strategic implementations, these wasted energies can be converted into useful sources to reduce the dependency on fossil fuels.

**Fig. 2:**
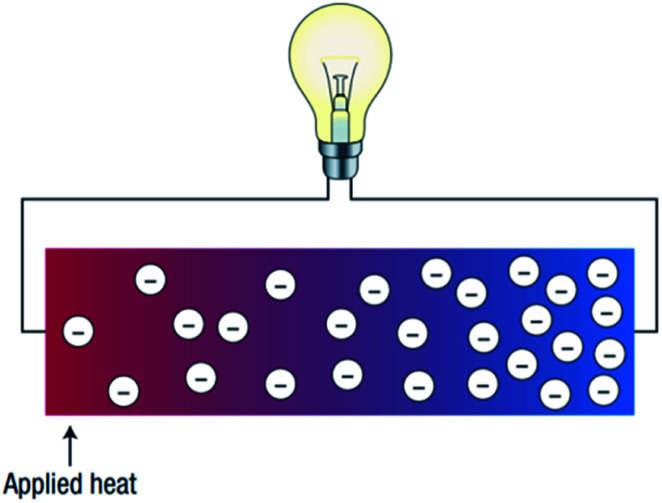
Schematic illustration of a thermoelectric device.^7,8^

**Fig. 3:**
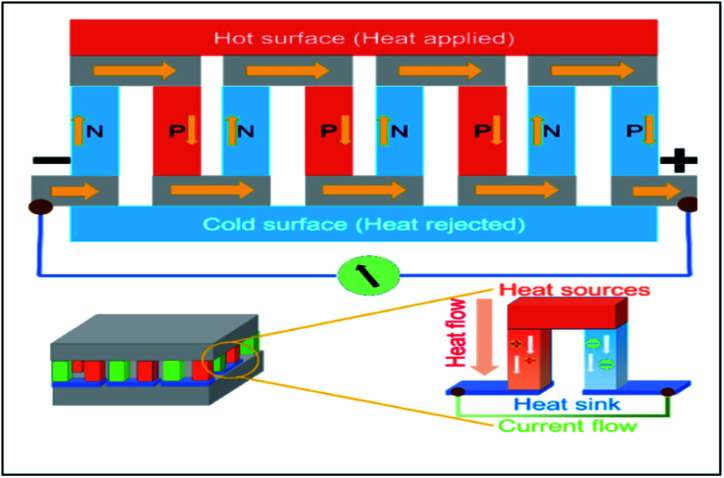
Schematic diagram for the assembly of thermoelectric devices.^9^

Thermoelectric devices are used to harvest thermal energy by directly recovering waste heat and converting it into useful electrical energy.^5,6^ The higher the amount of waste heat captured; the more the waste heat can be converted into electricity. Fig. 2 illustrates how thermoelectricity is generated. According to Fig. 2, the application of heat generates a temperature gradient along with the thermoelectric materials. Thus, a potential difference is generated, which leads to the generation of electrical energy, or in other words, the generation of thermal energy through the supply of electrical energy. Thermoelectric devices (Fig. 3) can be designed by exploiting the thermal and electrical properties to enable the heat to flow from the cold side to a hot side (refrigeration) to generate electricity.^7,8^ However, up to this stage, the efficiency of thermoelectric devices is still low (5–20%).^2,5^ Hence, the selection of compatible thermoelectric materials is vital in the development of sustainable thermoelectric devices.

The thermoelectric efficiency depends on the properties of the materials, whereby the performance of these materials can be determined using ‘figure of merit’ denoted by *ZT*. The materials with a higher figure of merit are recommended to be used in thermoelectric devices. The *ZT* can be expressed as *ZT* = (*S*^2^*σT*)*κ*^−1^, where *S* is the Seebeck coefficient (V K^−1^), *σ* (S m^−1^) is electrical conductivity, *κ* (W m^−1^ K^−1^) is thermal conductivity and *T* (K) is absolute temperature.^10–12^ The larger the *ZT* value, the more thermoelectric materials convert energy more effectively. Theoretically, *ZT* ≥ 1 generates conversion efficiency of >10 per cent.^2,13^ Besides *ZT*, other parameters such as chemical stability, thermal stability and electron mobility can also significantly determine the feasibility of a specific material in thermoelectric applications.

Among the reported thermoelectric materials, low-dimensional, nano-scaled materials have also received much attention from the research community. Significant *ZT* improvements have been found in various 1D/2D thermoelectric materials, including graphene, SiGe-based nanocomposites, SbTe- and PbTe-based nanostructures.^14^ Among the reported materials, zinc oxide (ZnO) is recognised as one of the promising thermoelectric materials.^15^ It has excellent physical and chemical properties including high chemical, thermal and mechanical stability, high electron mobility, corrosion resistance, degradable, easy for doping and synthesis.^16^ Moreover, ZnO is a cheap,^17^ safer and low toxic material,^7,18^ compared to lead telluride (PbTe) and tin telluride (SnTe).^12^ Other ZnO properties are given in Table 1. Despite these advantages, the major challenge of ZnO is that it has low electrical conductivity^19^ and high thermal conductivity of approximately 49 W m^−1^ K^−1^ at 300 K and 10 W m^−1^ K^−1^ at 1000 K (ref. 20) which limits the usage and efficiency of ZnO in thermoelectric applications. ZnO's excellent heat conductivity is explained by its simple crystal structure and light element composition. Hence, leading to a poor *ZT* value of less than 0.01.^13^

**Table 1:** Properties of ZnO

Properties	Values	References
Mineral	Zincite	21
Appearance	White powder	
Solubility	Soluble in water and alcohol	
Group	II–IV compound semiconductor material	10, 22 and 23
Crystal structure	Hexagonal wurtzite (Wz)	10 and 22
Lattice parameters	*a* and *c* (*a* = 3.249 and *c* = 5.206 Å)	10 and 22
Space group	*P*6_3_*mc* (186)	10 and 22
Band gap energy	3.3 eV at room temperature	23
	∼3.3–3.37 eV at 300 K	10
Exciton binding energy	60 meV at room temperature	24
*ZT* value	Poor *ZT* value (∼0.005)	25
Field emission	∼18 V mm^−1^ at a current density of 0.01 mA cm^−2^	26
Luminescence	The band gap of 3.37 eV, binding energy 60 meV	26

Numerous studies have tried many strategies to achieve higher performance factors and lower thermal conductivity in ZnO ceramics. The strategies include morphological control,^27^ ion implantation,^28^ defects introduction,^29^*etc.* Among the reported strategies, the morphological control technique has been demonstrated promising potentials as it correlated directly with the phonon scattering and lattice thermal conductivity of nanostructured materials.^30^ The thermal conductivity of ZnO ceramics can be fine-tuned by altering its grain size. However, only limited studies reported the effects of ZnO ceramic grain sizes on its thermal conductivity and thermoelectric properties. Therefore, the effects of synthesis parameters focusing on grain size on the thermoelectric effects of ZnO ceramics are discussed and compared in this paper.

## 2. Reduction in thermal conductivity value

**Fig. 4:**
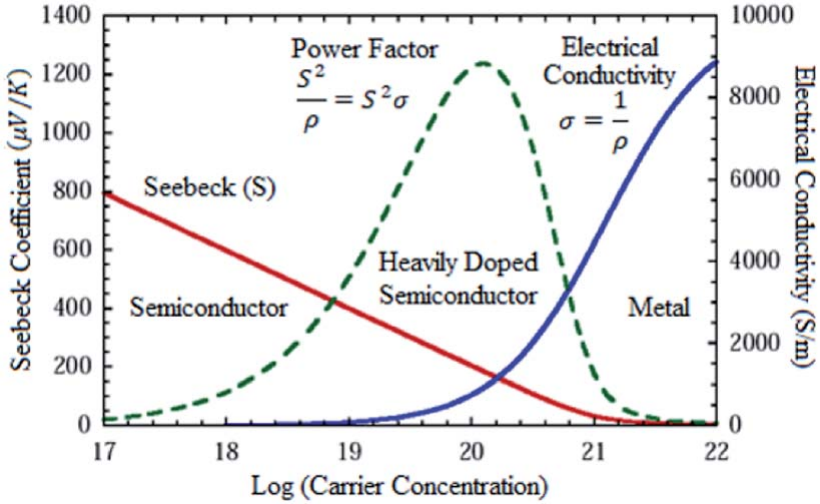
Seebeck coefficient, power factor and electrical conductivity *versus* log (carrier concentration).^18,31^

As mentioned earlier, the performance of thermoelectric materials can be mainly benchmarked using *ZT*, where *ZT* = (*S*^2^*σT*)*κ*^−1^. An excellent thermoelectric material exhibits a high Seebeck coefficient, high electrical conductivity and low thermal conductivity. A higher Seebeck coefficient value is required to obtain high voltage. While a higher electrical conductivity is required to minimize the Joule heating effect, reduce the internal resistance of the material and minimize ohmic losses. Meanwhile, a lower thermal conductivity is required in order to generate a large temperature difference.^2,11^ Improving the *ZT* value is quite a complex task since the parameters which govern the performance of thermoelectric materials are closely interrelated as an increment of one value will reduce the others.^11,12^Fig. 4 illustrates the relationship between Seebeck coefficient (red line), power factor (green dashed line) and electrical conductivity (blue line) *versus* carrier concentration on semiconductor, heavily doped semiconductor and metal. Based on the figure, metal exhibits high electrical conductivity with small Seebeck coefficient values, resulting in low power factor values.^18,31^

**Fig. 5:**
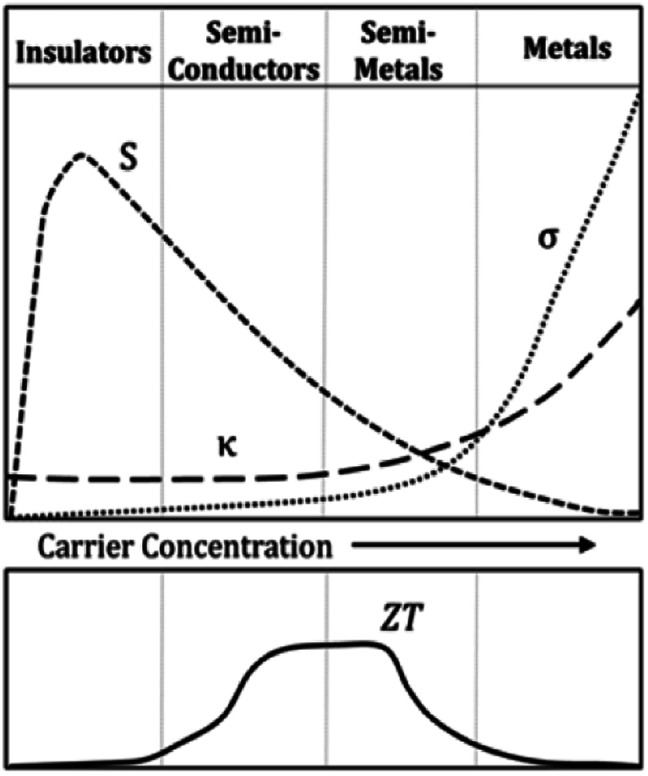
Thermoelectric behavior of different classes of materials.^32,33^

On the other hand, Fig. 5 shows the thermoelectric behavior of different material classes. The figure indicates that the insulators are poor thermal and electrical conductors.^32,33^ At the same time, semiconductors and semi-metals exhibited large thermopower, relatively high electrical conductivity and lower thermal conductivity which may lead to an optimum *ZT* value.^32,34^

However, improperly controlling these parameters (Seebeck, power factor and electrical conductivity) may cause difficulties in improving the *ZT* values. Some of the various control strategies to manage these parameters include doping,^35^ alloying^36^ and nanostructuring^37^ that attempt to obtain higher power factor and lower thermal conductivity. Following the nanostructuring approach, improvements have been performed to obtain high thermoelectric performance.^10^ Most of the nanostructuring research has focused on reducing the thermal conductivity value. In low-dimensional nanostructured materials (*e.g.*, ZnO ceramics), the thermoelectric properties of these materials can be enhanced by improving the Seeback coefficient and reducing thermal conductivity, as mentioned earlier. Nanoscale constituent-induced quantum confinement can be employed to improve the Seeback coefficient, whereas, enhanced phonon scattering by the interfacial structure surfaces in the nanostructures can be employed to reduce their thermal conductivity.^38^ These interfacial structure surfaces can be referred to as grain boundaries in nanostructured materials. The effects of grain boundary on thermal conductivity by phonon scattering has been described by Callaway,^39,40^ which is expressed as:1
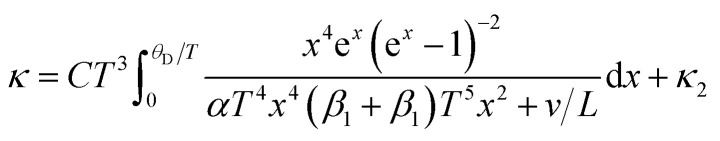
where *C* can be expressed as:2
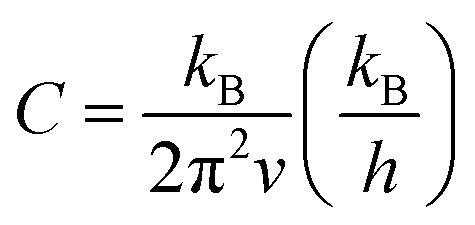
and *x* can be expressed as:3
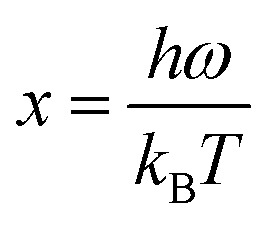
while *T* is absolute temperature, *θ*_D_ is Debye temperature, *v* is phonon velocity, *L* is a characteristic length of material, *ω* is circular frequency, *k*_B_ is Boltzmann constant and *h* is Planck constant. *κ*_2_ is a correction due to the conservative nature of the scattering process and is generally negligible in calculations. Based on the context of nanostructured materials, *L* would be represented by the grain size of the nanostructures. According to the *κ versus L* model, it can be deduced that smaller grain sizes (*L* values) will result in lower thermal conductivity (*κ*) in the nanostructure, resulting in improved thermoelectric properties.

The high thermal conductivity of ZnO will lead to poor *ZT* value. The high thermal conductivity of ZnO is due to the higher phonon frequency of the ionic bonding and lighter atomic mass.^41^ Fundamentally, thermal conductivity is the property of the material to conduct heat and is measured in watts per meter Kelvin (W m^−1^ K^−1^). The thermal conductivity is the sum of phonons and electronic charge carriers (electrons or holes as expressed as *κ* = *κ*_lat_ + *κ*_ele_ where *κ*_lat_ is lattice thermal conductivity and, *κ*_ele_ is electrical thermal conductivity).^11,42^ Since the power factor is correlated with *κ*_ele_, an increased *ZT* can be achieved when the thermal conductivity of the heat grille is reduced.^43^ The value of thermal conductivity can be decreased by increasing the phonon scattering at grain boundaries and refining the grain sizes.^41,44^ As described by Callaway, when the grain size is decreased to the nanoscale, the scattering rate of phonons become higher. Hence, it will lower the value of lattice thermal conductivity, which is favourable for thermoelectric materials. However, as thermal conductivity is reduced, the value of electron scattering increases to lower the value of power factor,^41,44^ resulting in drawbacks in thermoelectric properties.

Over the past decade, many studies focused on altering ZnO grain size to achieve lower thermal conductivity for optical thermoelectric performances.^45^ Kinemuchi *et al.*^46^ reported on the grain size dependency in thermal conductivity of ZnO clusters. In the reported study, the grain size of ZnO clusters can be moderated by fine-tuning the heating and pressure parameters during the synthesis process. As a result, ZnO clusters with smaller grain sizes exhibited lower thermal conductivity, which is consistent with the Callaway model.^47^ In addition to optimizing the synthesis process (heat treatment and application of pressure), another approach to controlling the grain size of ZnO is to inhibit foreign particles to suppress grain growth. According to Narjis *et al.*,^48^ the incorporation of aluminum (Al) with ZnO leads to gahnite–ZnAl_2_O_4_ precipitations, which indicates an inhibition of the ZnO grain growth.^47^ Therefore, by optimizing the Al incorporation and the processing parameters, a significant reduction in the thermal conductivity of ∼52% can be achieved in order to achieve an effective energy conversion in thermoelectric applications.^49^

The above techniques are mainly applied to the production of ZnO ceramics using the solid-state reaction process. However, the production of ZnO nanoparticles is a complex process that can be achieved using many techniques such as the sol–gel method, the solid-state reaction method, the solution method, microwave synthesis, liquid path synthesis, and others. Among the available synthesis methods, synthesizing ZnO ceramic *via* chemical route is preferred as it demonstrates lower synthesis costs and involves lower synthesis temperatures compared to its solid-state counterparts.^50^ Various factors in the chemical synthesis can influence the thermal conductivity namely synthesis method, temperature, pH and materials that affect the grain sizes. In this article, the review will focus on the relationship between the types of chemical synthesis methods, the parameters involved in the synthesis of ZnO and its corresponding grain sizes.

## 3. Effective parameters on ZnO grain sizes

### 3.1. Effects of chemical synthesis method on grain size and thermal conductivity

As discussed in the previous section, there are various ways of synthesizing ZnO ceramic. In the context of thermoelectric applications, the desired ZnO clusters with smaller grain boundaries correspond to lower thermal conductivity and high *ZT* values. Due to the differences in the process, different synthesis techniques produce ZnO ceramics of various grain sizes and thermal conductivity. Thus, the preferred synthesis method selected to produce ZnO-based thermoelectric materials will consider various factors, including obtained grain size, thermal conductivity, process complexity, fabrication costs, *etc.*

The relationship between grain size and thermal conductivity of zinc acetate dihydrate using nanostructuring and non-nanostructuring approaches are reviewed in this study. Fundamentally, nanostructuring can be described as creating materials composed of nanometer-size grains or with other nanometer-scale internal structures to alter the grain size and other microscopic properties.^51^ This approach is potentially in reducing the thermal conductivity in many thermoelectric material systems, thereby improving the thermoelectric performance.^52^ According to several studies, decreasing the grain size down to nanoscale (< 100 nm) can improve the thermoelectric performance.^42^

Table 2 summarizes the results of the grain size and thermal conductivity at 700 K using various chemical synthesis methods. It can be concluded that the ZnO grain sizes obtained from nanostructuring approaches are smaller than those obtained from non-nanostructuring approaches. Table 2 also showed that a smaller grain size, which results in lower thermal conductivity, fits well with the Callaways model, as discussed in the previous section. For instance, Baghdadi *et al.*'s^53^ Al-doped ZnO nanoparticles synthesized by microwave irradiation method yielded nanometer-sized ZnO. The obtained ZnO grain size sample was 24.1 nm, where its thermal conductivity measured at 700 K was ∼2.2 W m^−1^ K^−1^ and 4 W m^−1^ K^−1^ at room temperature. The thermal conductivity of nanostructured ZnO was reduced by ∼99% compared to 49 W m^−1^ K^−1^ for bulk ZnO samples. The obtained *ZT* value was ∼0.028 at 675 K, approximately 15 times higher compared to the non-nanostructuring approach. Hence, it can be deduced that the usage of microwave sintering systems yielded rapid heating cycles with low temperatures. Thus, resulting in anisotropic grain growth and rapid diffusivity of ZnO particles which suppresses the grain growth of ZnO during the synthesis process.^52^ Despite the advantages of the microwave irradiation method including small-grained and low thermal conductivity of ZnO, this method requires expensive and sophisticated equipment for its complex processes.

**Table 2:** Chemical synthesis method, grain size and thermal conductivity (W m^−1^ K^−1^), figure of merit (*ZT*) at 700 K of nanoparticles thermoelectric materials

No.	Chemical synthesis method	Approach	Materials	Grain size (nm)	Thermal conductivity, *κ* (W m^−1^ K^−1^), figure of merit (*ZT*) at 700 K	References
1	Microwave irradiation method	N	(Zn_0.98_Al_0.02_)O	24.1	∼2.2, *ZT* ∼ 0.03	53 and 55
2	Solution method	N	(Zn_0.98_Al_0.02_)O	∼90	∼3.2, *ZT* ∼ 0.08	42, 54–56
3	Sol–gel method	NN	ZnO	100–2000	∼11.3, *ZT* ∼ 0.0016	25, 57–59 and 70
			(Zn_0.95_Al_0.02_)O	100	∼10, *ZT* ∼ 0.15	
4	RF plasma processing technique	NN	ZnO	2520	∼12, *ZT* ∼ 0.002	54 and 60
			(Zn_0.98_Al_0.02_)O	850	∼13, *ZT* ∼ 0.01	
5	Solid-state reaction method	NN	(Zn_0.98_Bi_0.02_)O	2000–6000	∼15, *ZT* ∼ 0.05	61 and 62

On the other hand, apart from the microwave irradiation method, a cheaper alternative in producing ZnO thermoelectric material is the solution method. Hsu (2019) synthesized Al-doped ZnO nanograins using a solution method.^54^ Researchers found that ZnO samples were mostly obtained with an average grain size of ∼90 nm. Thermal conductivity value was then successfully reduced to 7.6 W m^−1^ K^−1^ at room temperature, and monotonically decreased with increasing temperature (*κ*_700 K_ is ∼3.2 W m^−1^ K^−1^ and *κ*_1000 K_ is <2.0 W m^−1^ K^−1^).

The *ZT* value, which was 0.34 at 1073 K, is a significant increase compared to the non-structuring approach. The low thermal conductivity was obtained due to increasing phonon scattering at nanograin boundaries and nano precipitates. This observation also aligned well with the Callaway model as mentioned earlier, where the thermal conductivity of particles reduced with smaller particle sizes. Meanwhile, Kinemuchi *et al.* (2010)^46^ reported that the thermal conductivity in a ZnO nanocomposite can reach approximately 5 W m^−1^ K^−1^ at high temperature as the grain size reduces to smaller than 100 nm.^41^ Therefore, controlling the grain size, nanoprecipitate and carrier concentration in the nanocomposite will strongly enhance *ZT* values.

Based on the aforementioned outcomes, it can be observed that the nanostructuring techniques produced ZnO with smaller grain sizes compared to their non-nanostructuring counterparts. However, among the different nanostructuring techniques, the microwave irradiation method yielded more ZnO with much smaller grain size (24.1 nm) with the highest *ZT* (∼3.0) than that of the other nanostructuring techniques, yielding the lowest thermal conductivity of 2.2 W m^−1^ K^−1^. This *ZT* value agreed with Das^47^ who found that decreasing the grain size is an effective way to enhance the *ZT*.

Meanwhile, synthesizing ZnO using the solution method produced ZnO grains of approximately ∼90 nm with thermal conductivity of ∼3.2 W m^−1^ K^−1^. Although the microwave irradiation method offers the smallest ZnO grain size with the lowest thermal conductivity (Table 2), the solution method provided a cheaper alternative in obtaining ZnO with compatible thermoelectric performances to that of the ZnO clusters synthesized by the microwave irradiation method. Besides that, the solution method provides a green versatile approach, where the usage of various solution types, synthesis parameters and processing conditionings can be flexibly tuned to obtain the desired thermoelectric performances. Thus, the next section will focus on state-of-the-art synthesizing ZnO thermoelectric materials using the solution method.

### 3.2. Effects of solvent on grain size and thermal conductivity

Synthesizing ZnO thermoelectric materials using the solution method provides an affordable yet versatile approach in fine-tuning the grain size and associated thermoelectric properties of ZnO. This approach is a cheaper alternative compared to the microwave-assisted hydrothermal method. Fundamentally, a typical preparation technique of ZnO using the solution method involves a mixture of Zn-containing composites into a solvent. When Zn particles are dissolved into the solvent, another solvent containing hydroxide-based particles is added drop-wise into the Zn solvent to form precipitation. The precipitates are then centrifugally washed several times with de-ionized water and organic solvent to form highly pure ZnO nanoparticles.^63^ A similar method of mixing the Zn-containing solvent and the hydroxide-containing solvent is also used for the synthesis of the ZnO film. For example, to deposit a thermoelectric ZnO film on the glass substrate, the pre-cleaned substrate is first immersed in a Zn-containing solution in order to adsorb several layers of zinc complex ions on the substrate. After removing loosely-bound layers of Zn using deionized water, the substrate is then immersed in hydroxide anions-containing solutions to form ZnO.^64^ Thus, it can be postulated that the solvent type, reaction condition and reaction time play significant roles in dictating the properties (*e.g.* grain size and thermal conductivity) of ZnO clusters/films.

**Fig. 6:**
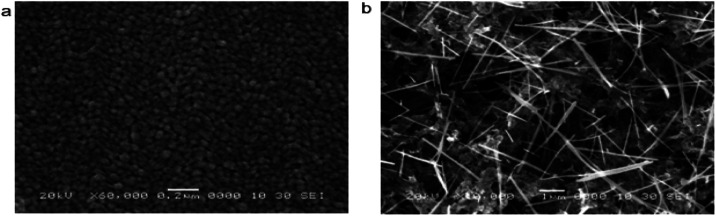
SEM images of the sample obtained by (a) NH_4_OH solvent; (b) NaOH solvent.^67,75,76^

Many studies have reported the influence of the solvent types on the growth of the ZnO grain size. In the current study, Table 3 represents the ZnO grain size on the usage of various solvents. The type of solvent that is important for the synthesis process of zinc acetate dihydrate also influences the particle size of ZnO powders. Moreover, Bari *et al.* (2009)^65^ reported that the grain size and particle size of nanostructured ZnO powder differ based on the usage of different solvents.^66^ In their study, the sol was prepared first by mixing zinc acetate with ethanol and ammonium hydroxide. The alkaline solution was then added to bring the ethanol to pH 7.2. Following that, polyethylene glycol (PEG) was added to obtain the gel. The procedures are repeated by adding sodium powder to vary the pH of the solvent. The varying pH of the solution was adjusted to 6.2 with sodium powder. All samples were annealed at 50 °C. The type of solvent affects the size and shape of the powder. When using ammonium hydroxide (NH_4_OH) as a solvent, grain sizes of 27 nm and 30 nm were observed; whereas for sodium powder (NaOH) solvents, the grain sizes were 39 nm and 60 nm.^67^ Based on the Scanning Electron Microscope (SEM) images in Fig. 6, the shape of the samples using NH_4_OH was spherical and for sodium hydroxide was tubular. Ammonia has higher pH (∼11) than diethylene glycol (∼9–10) resulted in lower thermal conductivity^68,69^ with a higher *ZT* value.

**Table 3:** Effects of solvent on the grain size (nm), particle size (nm), the crystallite size (nm), thermal conductivity and figure of merit of zinc acetate dihydrate (Zn(CH_3_COO)_2_·2H_2_O) using sol–gel method

No.	Solvent	Grain size (nm)	Crystallite size (nm)	Particle size (nm)	Thermal conductivity, *κ* (W m^−1^ K^−1^), figure of merit (*ZT*) at 700 K	References
1	Ammonia NH_3_H_2_O	100–2000	—	—	∼11.3, *ZT* ∼ 0.037	57 and 72
2	Diethylene glycol (C_4_H_10_O_2_)	—	—	—	∼13.42, *ZT* ∼ 0.012	72 and 73
3	Methanol (MeOH)	—	39–53	—	—	70
		—	18	—	—	74
		91.37	21.64	—	—	71
4	Ethanol (EtOH)	129.10	22.84	—	—	71
5	2-Methoxyethanol (2-ME)	58.14	19.12	—	—	71
6	Isopropyl alcohol (IPA)	87.91	21.19	—	—	71
7	Sodium powder (NaOH)	—	—	84.98	—	65
		39	—	60	—	67
8	Ammonium hydroxide (NH_4_OH)	27	—	30	—	67

Apart from the comparison between NH_4_OH and NaOH as mentioned above, Foo *et al.* (2013)^70^ synthesized ZnO thin films using a low-cost sol–gel spin-coating method with four different solvents namely methanol (MeOH), ethanol (EtOH), isopropyl alcohol (IPA) and 2-methoxy ethanol (2-ME).^71^ The grain size values were characterized using an atomic force microscope (AFM) while the average crystallite size of the ZnO thin films was estimated using Scherrer's formula. Based on the results, 2-ME yielded the smallest grain and crystallite sizes with 58.14 nm and 19.12 nm, followed by IPA, MeOH and EtOH with the grain size of 87.91 nm, 91.37 nm and 129.10 nm and crystallite size of 21.19 nm, 21.64 nm, and 22.84 nm, respectively. Hence, it can be concluded that the different solvents used affected the grain size and crystallite size.

Besides alkaline-based solvent introduced by Bari *et al.*^65,71^ and organic-based solvent by Piliai *et al.*^77^ another study reported on the synthesis of ZnO nanoparticles using acidic solvents. Kumar *et al.*^67,78^ reported the synthesis of ZnO particles using hydrofluoric acid (HF)-based solvents. In the reported work, ZnO powder was physically mixed with HF aqueous solution, with aluminum foil dipped into the solvent as a source of cations. Pre-cleaned glass substrates were dipped into the ZnO/Al/HF bath for ∼15 hours to enable ZnO nanoparticle precipitation and growth. The ZnO nanoparticles were grown *via* the formation of the zinc-fluoro complex (Al-doped ZnO nanofilms) from the reaction with aluminium metal ions (Al^+^). Moreover, by varying the concentrations of HF, ZnO grain sizes of 5–29 nm were obtained. Lower HF concentrations resulted in the formation of larger ZnO grain size, whereas higher HF concentrations formed smaller ZnO grain size. Apart from acting as a solvent and catalyzer in the formation of ZnO nanoclusters, HF is also an important nanostructuring ZnO. At lower HF concentrations in Al/ZnO/HF bath, the reaction rate for the formation of ZnO particles is slower, thus, reducing the evaporation rate leading to the formation of bulk crystallites.^79,80^

In short, Bari *et al.*^65^ compared the formation of ZnO grain size between two solvent types, NH_4_OH and NaOH.^71^ Foo *et al.*^70^ evaluated the size of ZnO grains in four organic solvents: MeOH, EtOH, IPA, and 2-ME.^81^ Meanwhile, Kumar *et al.*^67^ compared the ZnO grain size using HF solvents of various concentrations. Both Bari *et al.*^65^ and Kumar *et al.*^67^ employed solvent samples with varying pH values (ZnO and Al particles in NH_4_OH solvent with pH 7.2 and NaOH with a pH value of 6.2, respectively). On the other hand, Kumar *et al.*^67^ used HF solvents in various concentrations, therefore it was found that the pH values of HF solvents significantly changed the grain size of the ZnO particles. Nevertheless, the effects of solvent type and the pH values of solvents on ZnO grain size is still under-explored, hence, more investigations and experimental works are needed.

### 3.3. Effects of pH on grain size

As mentioned earlier, several factors which can be considered for selecting the suitable solvents for the synthesis of ZnO nanoparticles include solvent type, solvent concentration and solvent pH level. It has been reported that the ZnO structure is unable to be properly synthesized at pH 6 due to the high concentration of H + ions and low concentration of OH ions in the solvent.^82,83^ Since these ions may form H^+^ functional groups on the ZnO powders suspended in the solvent, these groups may result in inter-repulsive forces between ZnO particles, hindering the formation of ZnO nanograins.^84,85^ As for ZnO nanoparticles synthesized at neutral condition (pH 7), the concentration of H^+^ ions reacting with OH^−^ from NaOH is equivalent.^82,83^ Therefore, reduction in the pH, also reduces crystallization, further suggesting that higher pH exhibits a higher grain growth rate. In short, a change in pH leads to considerable changes in the shape and size of the particles.

**Fig. 7:**
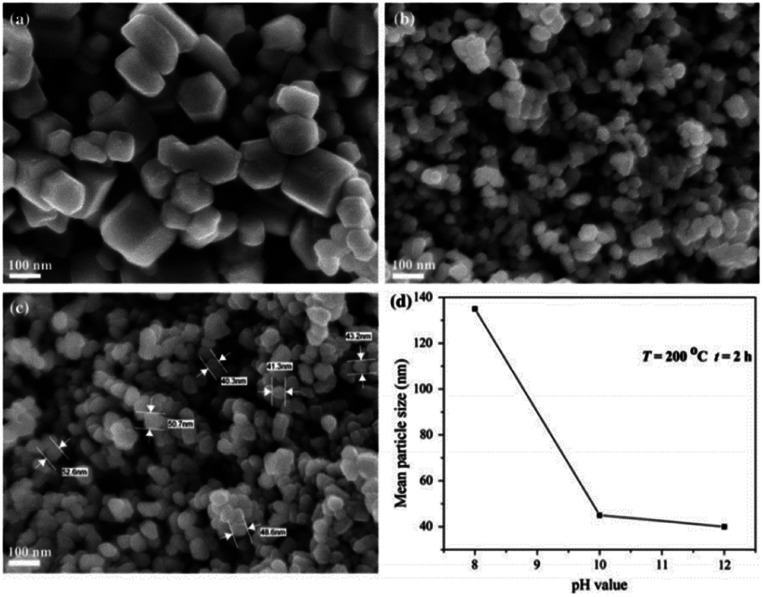
SEM images of ZnO powders obtained in solvothermal reactions temperature = 200 °C, reaction time = 2 h at: (a) pH = 8; (b) pH = 10; (c) pH = 12; (d) change in mean particle size of ZnO powders prepared by solvothermal synthesis with pH value of precursor solution.^84^

Luković Golić *et al.*^86^ demonstrated controlled morphology of micro- and nano-sized ZnO powders using variable pH levels of lithium hydroxide (LiOH) solution to examine the effects of the solvent's pH on ZnO grain size. Firstly, the Zn particles were dissolved in ethanol/zinc acetate dihydrate solution using the sol–gel method. The pH level of the solution was tuned by adding LiOH solution in deionized water. It was observed that in basic solutions, the increase of pH values led to a decrease in the particle size, forming a more uniform particle size. The SEM images and size distribution of ZnO is depicted in Fig. 7.^84^ Meanwhile, Chithra *et al.*^87^ reported similar outcomes, where the grain size of ZnO increased with an increasing pH level of the solvent.^88^ The study reported that the ZnO nanoparticles were synthesized using a simple precipitation method which involves Zn(CH_3_COOH)_2_·2H_2_O and NaOH. The solution was prepared using the ethanol solvent, where NaOH was added to vary the pH level of the solvent. The grain size of ZnO increased from 13.8 to 33 nm when the pH value of the solution was increased from 6 to 13.^89^

Based on the obtained outcomes, a basic solvent with a high pH favors the formation of small-grained ZnO nanoparticles. ZnO structure cannot be synthesized well at pH ≤ 6 due to the high concentration of H^+^ ions and a low concentration of OH^−^ ions in the solvent. In a nutshell, a higher pH alkaline solvent reduces the particle size to yield a more uniform particle size distribution. Table 4 summarizes the effects of pH on grain size (nm), particle size (nm) and crystallite size (nm). In the context of thermoelectric materials, ZnO nanoparticles with smaller grain sizes are associated with lower thermal conductivity and better thermoelectric performances. Therefore, the aforementioned examples can serve as a useful guide to synthesize ZnO thermoelectric particles for energy-conversion applications.

**Table 4:** Effects of pH on the grain size (nm), particle size (nm) and crystallite size (nm)

pH value	ZnO size (nm)			Shape	References
	Crystallite size	Particle size	Grain size		
2	23 nm	—	—	—	82
7	20 nm	—	—	—	82
8	24.96 nm	49.98 nm		Spherical	90
8.1	—	—	100–2000	Spherical (bulk)	57
9	25.36 nm	48.31 nm	—	Spherical	90
10	19 nm	—	—	—	82
10	21.87 nm	38.32 nm	—	—	90
11	18.37 nm	36.65 nm	—	—	90

### 3.4. The relationship between the particle size of the bulk sample and the thermal conductivity

**Fig. 8:**
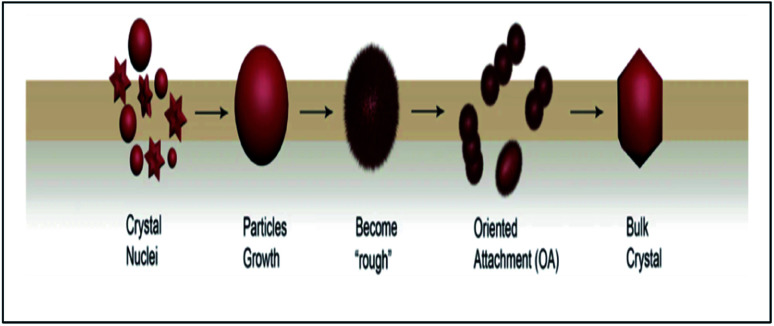
Stages of possible crystallization process-based OA.

ZnO nanoparticles tend to clump together, which is to be expected as the system tries to lower its total surface energy by adjusting crystal lattices and reducing exposed areas and defects. This typical process that occurs during nanoparticle growth eventually changes the particle surface structure.^91,92^ Based on the main points of the discussion above, the mechanism for oriented attachment (OA) during the formation of a suspension of ZnO NPs can be described, as shown in Fig. 8.

The formation of bulk nanoparticles undergoes three major stages:^92,93^

(i) Classical nucleation and crystal growth of the particles (nanocrystal formations);

(ii) Nanoparticles surface structure and morphology change (became the “rough” state);

(iii) Highly oriented aggregation between the nanoparticles (OA process).

If the growth state of the nanoparticles can be controlled in the coarse stage, the overall suspension maintains its dispersion for a long time according to this model. The development of crystal models for particle growth that are subject to similar mechanisms will improve synthetic strategies for nanomaterials.

Evans and co-workers concluded that the thermal conductivity of nanofluids was described by the effective medium theory, with the aggregation of the particles causing an improvement in the thermal conductivity of nanofluids.^94,95^ However, Wu *et al.*^96^ found that the size of 100 nm at room temperature can result in a decrease in thermal conductivity of ∼50% compared to massive crystals. This was due to the lower thermal conductivity of ZnO, which resulted from the smaller phonon group velocities, the larger three-phonon scattering phase space, and the larger anharmonicity.

### 3.5. Influence of ZnO particles on the thermoelectric (TE) performance

A reduction in the thermal conductivity of the lattice as a result of the scattering of phonons by nanostructuring^97,98^ was one of the mechanisms for improving the thermoelectric performance of materials.^98,99^ Studies showed that the grain size distribution influences the design parameters for the development of materials with superior properties and few researchers^100–102^ reported this can be done through inexpensive apparatus set to suit a wide range of applications. Another effective technique used to adjust nanostructure morphology/size of ZnO was using microwave irradiation and has been found by in providing highly efficient TE materials.^53^ The reduction in grain size leads to a decrease in thermal conductivity due to phonon scattering at the grain boundaries.^103^ A novel thermoelectric AI-doped ZnO ceramics with nanopowders size was proven enhanced TE performance through nano/microstructure engineered using synthesis technique.^25^

An average particle size of 6 nm of a thermoelectric ZnO thin-film sputter deposition with Ag nanoparticles opened up an effective way to improve the thermoelectric performance, especially in semiconducting layers.^104^ In addition, the ZnO nanoparticle size TE properties performance of cement paste was found. After adding the nanoparticles to the mixture, the thermal conductivity was reduced to 9% due to the lower density and crystallinity of the materials, and the electrical conductivity was increased to 37% compared to simple cement paste due to the increased ion movement.^105^

### 3.6. Effect of grain size on *ZT*

The influence of the grain size (*d*) on the thermal conductivity (*k*) of thermoelectric (TE) materials is well proven by experimental studies. Since the thermal conductivity makes a decisive contribution to the figure of merit (*ZT*) of thermoelectric materials, it is necessary to investigate the influence of the grain size distribution, an important microstructural descriptor, on *k*. The researchers found that reducing the grain size is an effective way to improve the *ZT*.^47,106^ The smallest grain size found can have the highest sometimes of ∼0.4 at 650 K.^107^ The smaller grain size is also decisive for the suppression of thermal conductivity through effective scattering of phonon waves.

## 4. Way forward

According to the aforementioned discussion, extensive experiments on the synthesis and optimization of ZnO nanoparticles for thermoelectric applications have been conducted. Fundamentally, small-grain ZnO with low thermal conductivity and high *ZT* values are desired for optimal thermoelectric performances. Several strategies have been investigated to accomplish this performance level, including the selection of synthesis procedures, solvent types, and fine-tuning of solvent pH levels to get the appropriate ZnO grain size and thermoelectric capabilities. Apart from that, the other approach that can be used is doping ZnO with foreign particles. It has been reported that doping ZnO with Al,^108^ graphene oxide,^109^ or gallium nitride^25^ improves the thermoelectric performance of ZnO by lowering its thermal conductivity through the synthesis of smaller ZnO nanograins.

Based on the literature review, many studies reported the optimization of ZnO thermoelectric performances. However, to apply these findings on an industrial scale, the experimental outcomes archived in the literature need to be standardized. Most of the published studies were carried out under various experimental conditions, namely obtained grain size, thermal conductivity and thermoelectric performances. Thus, there are no accurate or absolute values on the variations of ZnO's thermoelectric parameters with changing synthesis conditions, such as pH values, doping concentrations, synthesis temperature, *etc.* To realize the application of ZnO thermoelectric materials in energy conversion, standardization of ZnO properties using various experimental outcomes are crucial. It provides a firm foundation for research and engineering communities to construct ZnO-based thermoelectric devices.

## 5. Conclusions

This article presents a review of the research and development progress of the grain size effects in the ZnO thermoelectric materials. In this review, the figure of merit in obtaining ZnO with good thermoelectric performances was obtained, whereby low thermal conductivity and high *ZT* values are desired. According to the Callaway model, small-grained ZnO yield lower thermal conductivity, favoring its application in thermoelectric devices. Hence, several approaches have been discussed to reduce the grain size of ZnO as smaller grain size leads to a higher *ZT* value. Primarily, nanostructuring synthesis approaches are preferred over non-nanostructuring as they result in smaller grain sizes and lower thermal conductivity. Besides, the use of acidic, basic and organic solvents for ZnO synthesis was reported in varying grain sizes of ZnO nanoparticles. The pH level of solvent and its effects on the quality of ZnO nanoparticles formed has been discussed in this review. A higher pH level in acidic solution results in smaller, homogeneously-distributed ZnO grains favoring its application in thermoelectric devices. In conclusion, this review provided a comprehensive guideline on the effects of ZnO grain size in their thermoelectric performance along with the techniques to fine-tune the thermoelectric performances/grain sizes of ZnO. Hence, this review can be a useful preliminary guideline for future studies in optimizing the thermoelectric performances of ZnO nanoparticles.

## Conflicts of interest

There are no conflicts to declare.

## Supplementary Material
